# 2429 Optogenetic stimulation of corticotropin-releasing hormone expressing neurons in Barrington’s nucleus recapitulates the social stress voiding phenotype in mice

**DOI:** 10.1017/cts.2018.105

**Published:** 2018-11-21

**Authors:** Jason Van Batavia, Stephan Butler, Joanna Fesi, Rita Valentino, Stephen Zderic

**Affiliations:** 1 University of Pennsylvania School of Medicine; 2 Division of Urology, Children’s Hospital of Philadelphia; 3 National Institute on Drug Abuse, National Institutes of Health

## Abstract

OBJECTIVES/SPECIFIC AIMS: Voiding postponement is common cause of LUT dysfunction in which children void infrequently with large volumes. This condition is modeled in mice that are subjected to social stress who show decreased voiding frequency and increased voided volumes along with increases in corticotropin-releasing hormone (CRH) expression in Barrington’s nucleus (BN) (i.e., the pontine micturition center). Optogenetics is a technique to selectively stimulate cells or neurons of interest via light activated channel receptors [i.e., channel-2 rhodopsin (ChR2)]. Here we examined the effects of optogenetic manipulation of CRH BN neurons on the in vivo voiding phenotype and urodynamics in awake mice. We hypothesized that stimulating these neurons at higher frequencies (10–50 Hz) would lead to CRH-dependent alterations in voiding phenotype (i.e., larger voided volumes and longer intermicturition intervals. METHODS/STUDY POPULATION: Double transgenic mice expressing ChR2 in CRH cells were generated using the Cre-lox recombinase system and had fiberoptic probes implanted into BN at 8 weeks of age. The mice also underwent simultaneous catheter placement into the bladder for in vivo cystometry. In vivo cystometry before and during optogenetic stimulation at various frequencies was performed 5–7 days postoperatively. Saline was perfused at 10 µL/minute and baseline stable voiding cycles were established. Bladder capacity, threshold pressure, voiding pressure, and voided volume were recorded at baseline and at each optogenetic setting. In some mice, the protocol was repeated in the presence of CRH antagonist (NBI 30775). RESULTS/ANTICIPATED RESULTS: Fiberoptic stimulation (470 nm at 25 and 50 Hz) produced a significant rise in the intermicturition interval, bladder capacities and increased void volumes. This effect was especially pronounced in females in whom bladder capacity and intermicturition interval more than doubled at 50 Hz stimulation. Fluoroscopic images confirmed complete bladder emptying with each void. The increased bladder capacity at higher frequencies (25 and 50 Hz) was CRH-dependent as injection of a CRH antagonist (NBI 30775) blocked the optogenetic effect. Control non-double mice showed no effects from optogenetic stimulation. DISCUSSION/SIGNIFICANCE OF IMPACT: Our results suggest that optogenetic stimulation of CRH-expressing neurons in BN at high frequency (25 and 50 Hz) inhibits micturition and recapitulates the voiding phenotype seen in socially stressed mice (large, infrequent voids). Lower frequencies of optogenetic stimulation (2 and 10 Hz) had no effects on cystometry parameters or voiding phenotype. In addition, females had a greater response to optogenetic stimulation compared with males with larger bladder capacities and longer intermicturition intervals. The changes in voiding phenotype seen were CRH dependent as blockage of the CRH receptor prevented changes in cystometry parameters with optogenetic stimulation. Further elucidation of these and other neural subpopulations in BN are warranted to understand micturition and how it may be manipulated in disease states such as voiding postponement and acute urinary retention.

